# Rabies post-exposure prophylaxis delivery to ensure treatment efficacy and increase compliance

**DOI:** 10.1016/j.ijidoh.2023.100006

**Published:** 2023-12

**Authors:** Deborah Nadal, Katrin Bote, Ramesh Masthi, Ashwath Narayana, Yasmeen Ross, Ryan Wallace, Bernadette Abela

**Affiliations:** a Department of Humanities, Ca’ Foscari University of Venice: Malcanton Marcorà, Dorsoduro 3484/D, 30123 Venice, Italy; b Department of Control of Neglected Tropical Diseases, World Health Organization, Avenue Appia 20, 1202 Geneva, Switzerland; c Department of Community Medicine at the Kempegowda Institute of Medical Sciences Hospital & Research Centre, Krishna Rajendra Road, 560004 Bengaluru, Karnataka, India; d US Centers for Disease Control and Prevention, Clifton Road 1600, 30333 Atlanta, GA, United States

**Keywords:** Rabies post-exposure prophylaxis, Rabies vaccine, Rabies immunoglobulin, Intradermal vaccination, Wound treatment, Risk assessment

## Abstract

**Objectives:**

Since rabies is lethal once symptoms appear, its prevention including community awareness, mass dog vaccination and post-exposure prophylaxis (PEP) is crucial. Although safe and potent rabies vaccines have long been available, the global rabies burden is still high and access to adequately-delivered PEP remains challenging. Here we offer healthcare providers up-to-date, simple, exhaustive, visual guidance on how to perform PEP steps correctly.

**Protocol:**

PEP consists of 1) washing the wound with water and soap for 15 min, 2) assessing the need for rabies biologicals based on specific criteria; 3) administering, if required, rabies immunoglobulin or monoclonal antibodies deep in and around all wounds; 4) starting, if necessary, the WHO-recommended intradermal 1-week vaccination regimen; 5) informing patients adequately throughout the PEP process to increase compliance and avoid dangerous misconceptions about animal bite treatment and rabies risk.

**Discussion:**

The intradermal 1-week vaccination regimen recommended by WHO is as safe as other regimens but carries important cost-, dose- and time-sparing benefits. As fundamental as the correct administration of rabies biologicals is clear doctor-patient communication and sharing of up-to-date knowledge among healthcare professionals.

**Conclusions:**

This resource will help ensuring that no life is lost to rabies, an incurable yet preventable disease.

## Graphical Abstract

**Figure:**
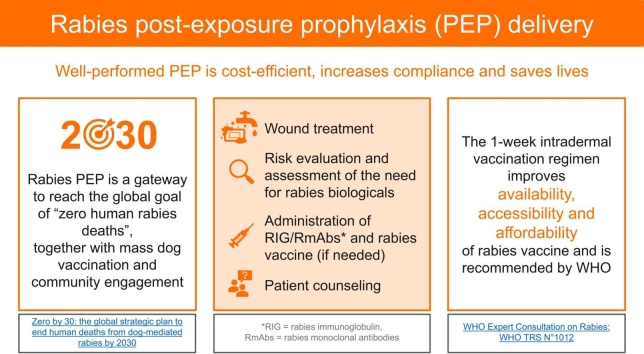

## 1 Introduction

Rabies is 100% fatal as soon as symptoms appear, but it is also 100% preventable by ensuring prompt access to and effective delivery of post-exposure prophylaxis (PEP), mass vaccinating rabies-susceptible dog populations, and increasing awareness and engagement in at-risk communities [1]. In 2018, the World Health Organization (WHO), the Food and Agriculture Organization of the United Nations, the World Organisation for Animal Health and the Global Alliance for Rabies Control set the global target of achieving zero human deaths from dog-mediated rabies by 2030 (“Zero by 30″) [2]. Most efforts need to focus on Africa and Asia, where the vast majority of the estimated 59,000 human rabies deaths occur each year, especially in socially, economically and marginalized settings [3].

Rabies mainly kills people who cannot access PEP timely or do not receive an appropriate regimen or effective biologicals [4], [5]. Therefore, it is critical to ensure, through awareness campaigns for communities and training sessions for healthcare providers, that all the available knowledge, resources, products and skills are put to the best use possible to save the lives of exposed individuals [6].

We hereby provide clinicians with a detailed protocol that guides them through the five components of a well-performed PEP delivery, and describes the rationale behind these recommendations, so they can appreciate their importance and explain it to their patients. The five components are: (1) wound treatment; (2) risk evaluation and assessment of the need for rabies biologicals; (3) if indicated, administration of rabies immunoglobulin (RIG) or rabies monoclonal antibodies (RmAbs); (4) if indicated, administration of rabies vaccination; (5) patient counselling, which encompasses all PEP delivery components.

## 2 Protocol

### 2.1 Wound treatment

*Thorough wound washing is a life-saving measure to mechanically reduce the viral inoculum at the wound site and is crucial in all exposures but especially when the patient’s trip to the healthcare facility is long or no rabies biologicals are readily available*.

i) Wash and flush the wound (or all wounds, if more than one) with copious amounts of water (preferably running water, if available) and soap or detergent for 15 min.

If eyes or mucosa were exposed, thoroughly rinse them with water.

ii) Apply an antiseptic (e.g., povidone-iodine) thoroughly on the wound.
iii) Since the wound is likely contaminated with dirt or soil, administer tetanus vaccination to the patient, either as a primary series or a booster if the last dose was received more than 10 years before (or as per the national vaccination guidelines). Consider broad-spectrum antibiotics to prevent bacterial infection especially in deep wounds, and follow the dosage recommended by the manufacturer.
iv) Advise the patient against applying home-based remedies to the wound.
v) Do not close or tightly cover the wound with dressings or bandages and advise the patient against doing this.
vi) Avoid suturing. If not possible (e.g., continued bleeding, risk of visible scar), try to delay it at least for some hours after the administration of RIG/RmAbs (if this rabies biological is necessary) to allow its infiltration through the tissues. If suturing cannot be delayed, sutures should be loose and minimal.

### 2.2 Risk evaluation and assessment of the need for rabies biologicals

i) Check the severity of the wound.

Assess the number of wounds and their depth, and determine the WHO wound category [1]:

• Category I: The skin is still intact (test: the patient feels no burning sensation when surgical spirit is applied on intact skin): animal licks on intact skin, touching or feeding animals. This is not an exposure.
• Category II: The skin is broken but there is no bleeding (test: the patient feels a burning sensation when surgical spirit is applied on broken skin): minor scratches or abrasions, nibbling of uncovered skin. This is a mild exposure.
• Category III: The skin is broken and the wound is bleeding, mucous membrane or broken skin has been contaminated with saliva or direct contact with wild animals (including bats) has occurred. This is a severe exposure.

ii) Consider the anatomical position of the wound.

Assess if it is a wound to a body part that is close to the brain or highly innervated, hence making it easier and faster for the virus to reach the brain. Such high-risk areas are the head, neck, face, genitals, or hands.

iii) Assess if the patient is immunodeficient.

Ask the patient whether they suffer from any immune system disorder, or are HIV positive and on any anti-retroviral treatment, or are on long-term steroids or anti-cancer drugs.

iv) Assess the vaccination history of the patient.

Ask the patient whether they have ever received any rabies vaccination (preferably with proof of vaccination, e.g., vaccination card), either before any exposure (as pre-exposure prophylaxis, PrEP) or after any exposure (as PEP).

If the patient has ever received any rabies vaccination before, ask the patient how many doses they have received. At least two doses of a cell culture vaccine received on an appropriate schedule before discontinuation count as PrEP. If the patient cannot remember the number of doses received or vaccination dates, or cannot show proof of prior vaccination, consider them unvaccinated patients.

If the patient has ever received any rabies vaccination before, ask the patient when they have received these doses. If complete PEP has been received in the last 3 months, immediate vaccination is not recommended. Since the patient might not know for sure whether they received the rabies vaccine, or another vaccine, proof of prior vaccination is recommended.

If the patient has ever received any rabies vaccination before, ask the patient whether they had any adverse events after vaccination. If the patient had a mild local reaction (e.g., pain, redness or swelling), they can continue PEP using the same brand of rabies vaccine. If they had a severe local or systemic reaction, another type of rabies vaccine should be used, if available (e.g., purified chick embryo cell vaccine, purified vero cell rabies vaccine).

v) Gather information from the patient on the animal involved in the exposure.

Ask the patient what animal they were exposed to and determine whether it’s a rabies-susceptible animal. Only mammals are susceptible to rabies, although to different extents (e.g., rabies is rare in rodents, and no rodent bite is known to have caused a human rabies death).

**Figure 1:**
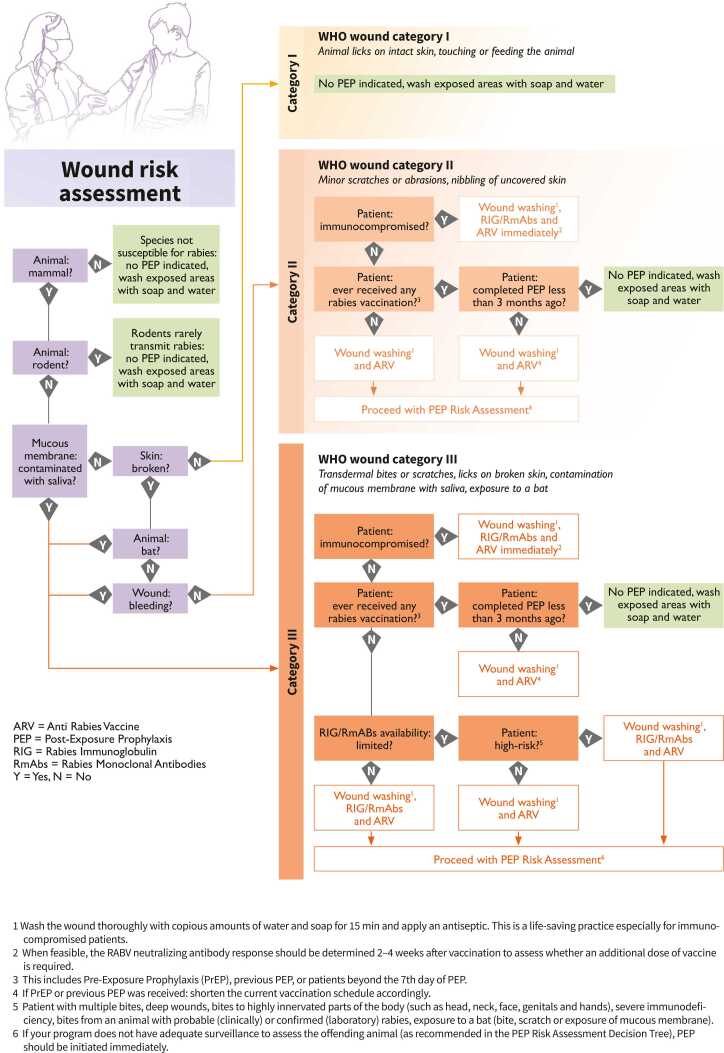
Post-exposure prophylaxis decision tree 1: wound risk assessment. This decision tree compiles all needed information on the assessment of the patient’s wound to guide healthcare providers on the appropriate, WHO-recommended PEP delivery.

**Figure 2:**
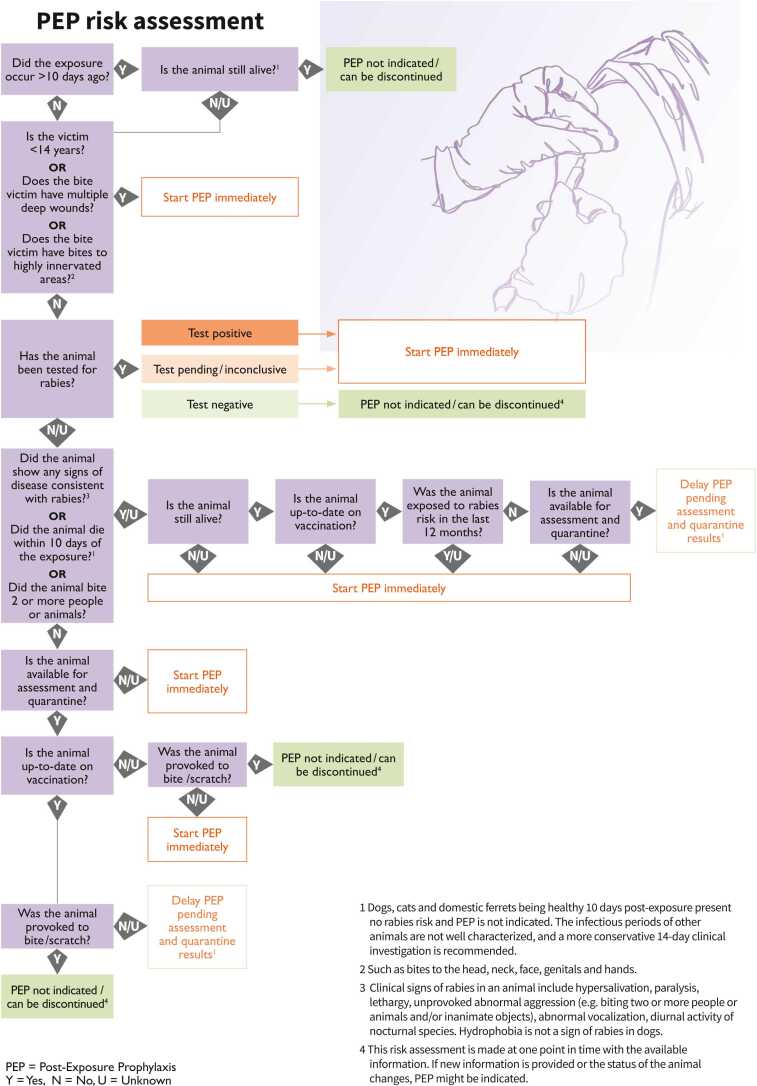
Post-exposure prophylaxis decision tree 2: PEP risk assessment. This decision tree compiles all needed information on the assessment of the animal exposure to guide healthcare providers on the appropriate, WHO-recommended PEP delivery.
vii) Fully and clearly explain to the patient the provided treatment (including any injection other than rabies biologicals, to avoid confusion in the patient) and the proposed rabies vaccination schedule, specifying how many visits and injections will be necessary (Fig. 3). Whenever possible, use the local language and choose words that are understandable to illiterate patients.

Ask the patient to give you any further information about the animal and the exposure to it (Table 1).

vi) Use all the information obtained to decide whether rabies vaccines and RIG/RmAbs are needed right away, can be delayed or are not applicable. For guidance on wound categorization and assessing the animal status, decision trees can be consulted (Fig. 1 and Fig. 2).

**Figure 3:**
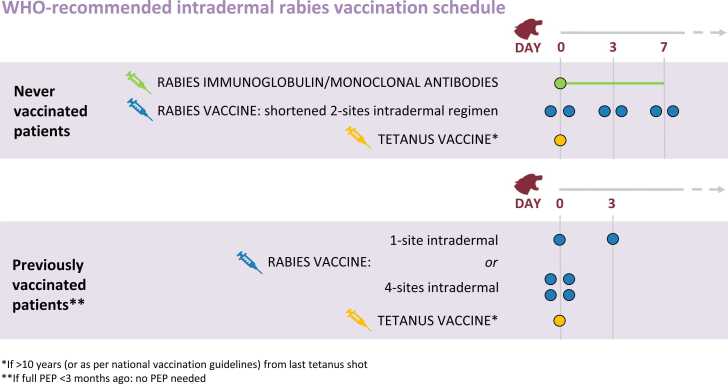
WHO-recommended 1-week intradermal post-exposure prophylaxis vaccination schedule. For exposed individuals who have not been previously immunized, WHO recommends a 1-week vaccination schedule on days 0, 3 and 7, with a 2-site intradermal injection on each day.
viii) Obtain consent to proceed as per local law and regulations.

**Table 1:** Questions on the animal involved to ask patients.

When did exposure occur?
Is the animal still alive? If not, when did it die?
Was the animal tested for rabies? If yes, what is the result of the test? If no, is the animal available for testing or quarantine?
Did the animal show symptomps like: Hypersalivation? Paralysis? Lethargy? Abnormal aggression (e.g., biting two or more people or animals and/or inanimate objects)? Strange vocalization? Diurnal activity (in case of nocturnal species)?
Do you know for sure whether the animal was vaccinated against rabies?
How did the bite/scratch/exposure happen? What was the animal doing and what were you doing?
Has the animal scratched/bitten other people/animals? If yes, inform them about the need for PEP, through local human and animal healthcare providers.

### 2.3 Administration of RIG/RmAbs

*The timely administration of RIG or any approved RmAbs provides passive immunization by neutralizing the rabies virus at the wound site before the immune system responds to the vaccine. Therefore, the products need to be administered into and around all wounds. RIG is derived from human blood (hRIG) or equine blood (eRIG). Strong evidence shows that both RIG versions have similar efficacy**[7]**. In clinical trials, several RmAb products against rabies have proved to be safe and effective in neutralizing a broad panel of globally prevalent rabies virus isolates. Advantages of RmAb products include large-scale production with standardized quality, high effectiveness and reduced risk of adverse events. RIG and RmAb products that are licensed for use in humans should be prioritized. WHO has recommended use of mAb “cocktails” containing at least two antibodies against the rabies virus**[1]*.

i) Take the RIG/RmAb box out of a properly functioning refrigerator.
ii) Calculate the maximum amount of RIG/RmAbs that the patient could receive: 20 IU (international unit)/kg of body weight for hRIG, 40 IU/kg of body weight for eRIG, 3.33 IU/kg of body weight for single RmAb and 40 IU/kg body weight for cocktail RmAbs.

If the wound is small, estimate the maximal quantity that is anatomically feasible to infiltrate and ensure not to exceed it.

iii) Draw the needed amount of RIG/RmAbs into a new syringe.
iv) Fractionate the rest into smaller, individual syringes to be used for other patients.
v) To thoroughly infiltrate large and/or multiple wounds, dilute RIG/RmAbs with the appropriate diluent (saline or 5% dextrose in water) based on manufacturer recommendations.
vi) For RIG: infiltrate the entire necessary amount or as much as possible carefully deep into or as close as possible to all wound(s) or exposure sites, avoiding any compartment syndrome. Injecting the remaining RIG volume intramuscularly at a distance from the wound provides no additional protection against rabies (see exception below). For RmAbs: follow the manufacturer’s instructions.

If there is a high likelihood that there are additional small wounds (e.g., if a child does not report all wounds), exposure was to bats, or exposure was other than through a scratch or bite, inject the remaining RIG volume intramuscularly as close as possible to the presumed exposure site, to the degree that is anatomically feasible.

If exposure was in the mucosa, rinse the exposed part with RIG.

If exposure was via aerosols (e.g., in a laboratory), inject RIG intramuscularly.

vii) Observe the patient for 15–20 min after the administration for potential adverse events, while you continue with the next steps. ERIG should be administered under conditions that allow the management of an anaphylactic reaction (i.e., a severe, potentially life-threatening allergic reaction to a substance such as a vaccine component). Nevertheless, the risk for an anaphylactic reaction is low (1/150,000), and the reaction is generally treatable.
viii) Store open vials and draw up doses (using new needles) if other patients come in on the same day.
ix) Discard open vials and syringes containing unused doses at the end of the day.

### 2.4 Administration of rabies vaccine

*Since 1984, WHO has strongly suggested the discontinuation of old-fashioned nerve tissue vaccines and their replacement with modern, concentrated, purified cell culture and embryonated egg-based rabies vaccines (CCEEVs)**[1]*. *Modern rabies CCEEVs are safe and well tolerated and should comply with the recommended potency of ≥ 2.5 IU per vial. All vaccines can be used both for intramuscular administration (i.e., injection in the muscle) and intradermal administration (i.e., injection in the upper layer of the skin). For dose-, cost- and time-saving reasons*[8], [9], *WHO recommends intradermal administration in a shortened, 1-week vaccination schedule (see*Fig. 3*above)**[10]*. *Rabies vaccines are safe to be used intradermally even when they are only labelled for intramuscular use (off-label use).*

i) If available, use WHO-prequalified cell culture vaccines (an updated list is available on the WHO website [11]). If WHO-prequalified cell culture vaccines are not available, use the cell culture vaccines recommended in national guidelines.
ii) Take the rabies vaccine box out of a properly functioning refrigerator. Rabies vaccines must be refrigerated at 2–8 °C, kept away from sunlight, and not stored in the refrigerator door (because the temperature may fluctuate when opening and closing it). The temperature of the refrigerator needs to be monitored and adjusted as necessary, especially in case of power cuts.

If an unopened vial is used, reconstitute the rabies vaccine according to the manufacturer’s instructions. All rabies vaccines are lyophilized and need reconstitution before use with the accompanying sterile diluent. Shake the vial 2–3 times before withdrawing the vaccine into the syringe.

If an already opened vial is used, check if it was stored hygienically and opened less than 6–8 h before. No need for reconstitution is there. Rabies vaccines must be used immediately after dilution, or within 6–8 h only if stored at 2–8 °C and protected from sunlight. Shake the vial 2–3 times before withdrawing the vaccine into the syringe.

iii) Draw up 0.2 mL of rabies vaccine with an insulin syringe.
iv) Inject 0.1 mL of vaccine intradermally in the deltoid area for adults and the anterolateral area of the thighs for children aged < 2 years. Rabies vaccines should never be administered in the gluteal area because this results in lower neutralizing antibody titres and should never be administered in the same anatomical site as RIG/RmAbs.

Insert the needle into the upper layer of the skin, with the bevel facing upwards, at a 45-degree angle and approximately 2 mm into the skin (similar to the Mantoux tuberculin skin test [12]).

Start injecting and notice whether you feel any resistance. If not, the needle may wrongly be in the subcutaneous tissue. In this case, withdraw the needle and repeat the injection in a new site.

**Figure 4:**
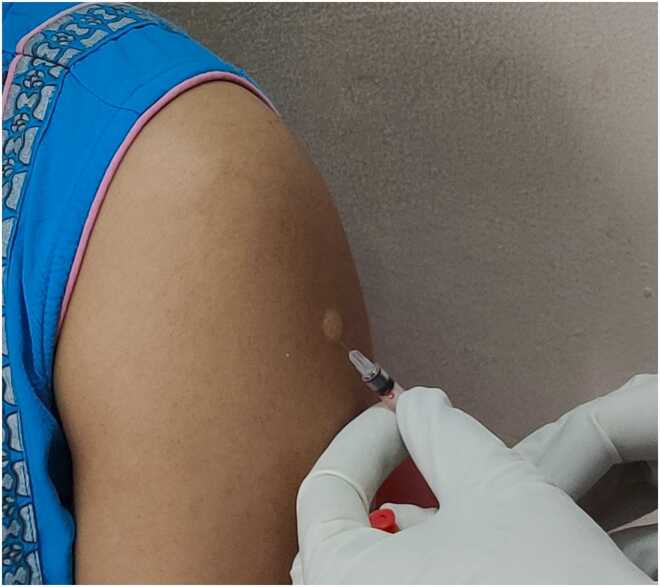
Confirmation of correct intradermal rabies vaccine administration. When the rabies vaccine is correctly injected into the upper skin area (over the deltoid muscle in adults or the lateral thigh area of infants), a distinctive bleb with an ‘orange peel’ appearance is visible.

Inject 0.1 mL until you see a small (i.e., 6–8 mm in diameter) bleb with an “orange peel” appearance (Fig. 4).

Do not rub the injection site.

v) Repeat the same on the other arm (or other thigh in children aged <2 years).

If the wound(s) is on an arm, the vaccine should be administered intradermally in the anterolateral area of the thighs or the suprascapular areas.

vi) Safely dispose of the used syringe and needle.
vii) Observe the patient for 15–20 min after the administration for potential adverse events.
viii) Store the opened vial hygienically.
ix) Hand out a rabies-specific vaccination card and remind the patient about the follow-up visits to complete PEP.
x) If the patient knows of any other person who was exposed to the same animal, ask the patient to promptly inform them about where to go to receive PEP.
xi) Register the information about exposure and treatment for national surveillance. The number of reported animal exposure cases in humans and the number of people receiving PEP (disaggregated by sex, age, species of exposed animal, and WHO exposure category) are key rabies indicators that need to be part of any national rabies surveillance systems.
xii) After 6–8 h from opening the vial, dispose of it.

### 2.5 Patient counselling

Get ready to answer the questions that your patients may have (Table 2A).

**Table 2:** Questions frequently asked by patients (2 A) and healthcare providers (2B).

Category	Question	Answer
**(A)**	I was scratched/bitten a long time ago. Do I still need rabies vaccination?	Yes, because the rabies incubation period can be very long.
	If I start rabies vaccination, will I need to change my diet?	No, you can eat whatever you want.
	Will you give me many injections in the stomach?	No, that was done with the outdated nerve tissue vaccine (NTV). Modern vaccines only require a few doses and a normal injection in the arm, as with any other injectable vaccine.
	Will there be adverse effects?	As with any vaccination, there may be adverse effects. But they are likely to be minor (such as redness, pain or swelling at the site of injection) or, even unlikely, mild (such as some fever, headache, dizziness or gastrointestinal symptoms). Serious adverse effects like allergic reactions are rare.
	What happens if I forget to come for the next vaccination?	Make sure you don’t forget by having your relatives/friends remind you about it. But if you forget, come as soon as possible and we will continue the vaccination, not restart it.
	I had milk from a rabid animal. Do I need rabies vaccination?	No, you don’t need any PEP, but avoid it next time and, anyway, milk should best be boiled before consumption.
	I had meat from a rabid animal. Do I need rabies vaccination?	No, you don’t need any PEP, but avoid it next time and, anyway, meat should best be cooked before consumption.
	I processed the meat of a rabid animal. Do I need rabies vaccination?	Probably yes. Tell me more.
	I was bitten by a mouse/small rat. Do I need rabies vaccination?	No, there is no risk of rabies.
	I am pregnant? Is rabies vaccination safe for me and my baby?	Yes.
	I am breastfeeding? Is rabies vaccination safe for my baby?	Yes.

**(B)**	Does the dose of rabies vaccine depend on age or weight?	No. Age only determines the site of rabies vaccination: the deltoid area for adults and the anterolateral area of the thighs for children < 2 years. Weight only determines the maximum amount of RIG/RmAbs to use: 20 IU/kg of body weight for hRIG, 40 IU/kg of body weight for eRIG, 3.33 IU/kg body weight for single mAb and 40 IU/kg body weight for cocktail mAb.
	Can I change the administration route or vaccine product during the vaccination schedule?	Yes, if unavoidable, you can do it. Don’t restart vaccination, just continue it.
	How does intradermal vaccination work when the dose is so small?	The antigen-presenting cells in the dermis are more effective in presenting the vaccine/antigen to the immune system than the ones in the muscles, so they can stimulate a very strong and prompt immunologic/antibody response.
	The rabies vaccine box only mentions intramuscular vaccination. Can I administer the vaccine intradermally?	Yes, all vaccines can be given both intramuscularly and intradermally, but choose the intradermal route whenever possible because it’s cheaper for the healthcare system and the patient, requires fewer visits, and one vaccine vial can be shared across many patients. However, check the rabies vaccines that your national drug regulatory authority approves for intradermal use.
	Can I inject the rabies vaccine in the gluteal area?	No, the vaccine would not be fully absorbed and effective because of the fat present in that body part.
	If the wound(s) is on an arm, where should I inject the rabies vaccine?	You should inject the vaccine intradermally in the anterolateral area of the thighs or the suprascapular areas. RIG must be injected in the wound(s).
	If RIG/RmAbs is not available on day 0, should I delay rabies vaccination?	No, never. But refer the patient to a healthcare facility where RIG/RmAbs is available, after administering the first dose of rabies vaccine.
	Can I administer RIG/RmAbs later on in the vaccination schedule?	Yes if RIG/RmAbs is not available on day 0, but never after day 7. Anyway, RIG/RmAbs should be given as soon as possible after exposure.
	Can I give RIG/RmAbs to a patient who has already received any rabies vaccination in their lifetime?	No, thanks to the previous vaccination, there are already demonstrable antibody titres or immune memory cells. In case of re-exposure, 1-site intradermal rabies vaccine administration on days 0 and 3 *or* 4-sites intradermal rabies vaccine administration on day 0 will produce good antibodies due to anamnestic response.
	Should I perform a skin test before administering eRIG?	No, because they poorly predict severe adverse events and their results must anyway not be the reason for not giving eRIG if it is needed. However, all RIG should be administered under conditions that would allow management of an anaphylactic reaction.
	Can I give rabies biologicals to a patient who is receiving treatment with chloroquine or hydroxychloroquine?	Yes, given the fatal outcome of rabies, there is no contraindication to the concomitant use of any medication.
	Can I give rabies biologicals to a patient who is receiving other vaccines in this period?	Yes, given the fatal outcome of rabies, priority is given to rabies biologicals (rabies vaccine and RIG/RmAbs). If the patient receives RIG, live vaccines should be postponed for 3–4 months, if possible.
	Should I perform an antibody test on the patient following rabies vaccination?	No, unless the patient is immunocompromised. In this case, a Rapid Fluorescent Foci Inhibition Test (RFFIT) or a Fluorescent Antibody Virus Neutralization (FAVN) test should be performed 2–4 weeks after vaccination to assess whether an additional vaccine administration is needed. Consultation with an infectious disease specialist or an immunologist is advised.
	Are there special recommendations for patients undergoing chemotherapy?	Yes, they are to be treated as immunocompromised patients. So: emphasis on proper wound washing; immediate RIG/RmAbs and rabies vaccine, even if previously immunized, for category-II and -III exposure; complete rabies vaccination course; Rapid Fluorescent Foci Inhibition Test 2–4 weeks after vaccination.
	Are all HIV-infected individuals considered immunocompromised?	No. HIV-infected individuals who receive antiretroviral therapy and are clinically well and immunologically stable (i.e., normal CD4% > 25% for children aged < 5 years or CD4 cell-count ≥ 200 cells/mm³ if aged ≥5 years) are not considered immunocompromised.
	Can intradermal administration be used for immunocompromised individuals or individuals receiving chloroquine, hydroxychloroquine drugs or long-term corticosteroid or other immunosuppressive therapy?	Yes.

Get ready to answer the questions that the less experienced colleagues may have (Table 2B).

## 3 Discussion

The PEP protocol is rather simple to follow, yet the following steps may present some challenges, particularly in healthcare settings that do not routinely provide treatment to persons with suspected rabies exposures.

The patient may have already cleaned or washed the wound at home, but most likely not to a sufficient extent. Mechanically removing the potentially infected saliva from the wound site is the number one first aid and it is especially essential for immunocompromised patients, who may not adequately respond to vaccination [10]. It is of utmost importance to raise community awareness about washing the wound immediately after exposure, wherever water (and possibly soap) is available, before seeking medical assistance. At the healthcare facility, an ad-hoc area for wound washing should be built to allow the comfortable washing of any body parts (e.g., leg, back, etc.).

After wound cleaning, the wound should ideally be left open and not sutured. If suturing cannot be avoided, the wound should first be thoroughly infiltrated with RIG/RmAbs (if necessary) and suturing should be delayed for several hours for RIG/RmAbs to spread through the tissues. Sutures should anyway be loose and as few as possible and, in case of risk of functional and/or aesthetic damage, it should be performed by a specialist [1].

A thorough PEP risk assessment is crucial to decide what is best for the single patient and, in case of limited availability of rabies biologicals, for other prospective, at-risk patients as well. PEP should start immediately, but only when necessary, to avoid resource wasting and overloading with patients who are not at risk. As indicated in the PEP decision trees (see Fig. 1 and Fig. 2 above), patients with severe immunodeficiency, patients with exposure on the head, neck, face, genitals or hands, young children, patients exposed to a bat or a clinically- or laboratory-diagnosed rabid animals and patients with multiple severe wounds are a high priority to start rabies vaccination immediately and receive RIG/RmAbs (as long as they have had a category II or III exposure to a rabies susceptible animal) [1]. This risk categorization is especially helpful in situations where RIG availability is limited [5].

Consultation and information sharing with an animal health expert (e.g., veterinarian) are key to further perfecting the risk assessment and deciding whether PEP is not indicated, can be delayed or can be discontinued (see Fig. 2 above). This animal risk assessment is based on the professionally-assessed health status of the animal involved, the possibility to observe it for 10 days (in dogs, cats and ferrets) or to test it, its reliable anti-rabies vaccination status and the events that led to exposure. If a patient comes in days, weeks or months after exposure, the case should be treated as if it was a fresh wound unless the animal was still alive 10 days (for dogs, cats and domestic ferrets) or 14 days (for other animals) after the exposure, as in this case the animal was not rabid. The step-by-step decision tree explained in Fig. 2 (above) covers all foreseeable scenarios and, if followed closely and in the suggested order, it is meant to avoid unfortunate and potentially risky situations where rabies biologicals are overused or underused. Moreover, this decision tree includes not only the answer options “Yes” and “No”, but also “Unknown”, because of the many possible uncertainties related to the animal the patient was exposed to (e.g., it has disappeared so it is not possible to know whether it’s still alive). That said, if in an area there is no adequate surveillance to assess the offending animal according to Fig. 2, PEP should be initiated immediately.

Identifying and following up on the animal involved is important also to recommend PEP to any other person, or animal, who may unknowingly be at risk and to remove dangerous and potentially rabid animals from the community. A strong surveillance system and One Health collaboration are necessary to jointly assess risk factors on both the patient side (see Fig. 1 above) and the offending animal side (see Fig. 2 above) [13]. Additionally, connecting human exposures to rabies susceptible animals, human rabies cases and animal rabies cases can help draft local endemicity maps, to better determine the level of rabies risk and deliver rabies biologicals in a more informed manner [14]. The setting up of an Integrated Bite Case Management system, which brings all the benefits just explained, is recommended to strengthen and institutionalize the collaboration between the human and animal health sectors.

Integrated Bite Case Management (or IBCM) is an advanced surveillance method that connects veterinary professionals, human health professionals and communities. It involves 1) conducting investigations of potential exposures and suspect rabid animals, 2) safely capturing the animal and assessing its health status, 3) keeping the animal under observation for 10 days (for dogs, cats and domestic ferrets) or 14 days (for other animals), and 4) sharing information with both animal and human health investigators for appropriate risk assessments to inform PEP decisions. IBCM programs are resource intensive but they ultimately help to prevent human rabies deaths (both by delivering PEP to current victims and avoiding further exposure through the removal of rabid animals from communities) and can improve the overall quality of surveillance.

The use of RIG has been hindered by concerns due to batch-to-batch variation in effectiveness, safety and quality, its short shelf-life (approximately 2 years) even with correct maintenance of cold chain (2–8 °C) and hesitancy regarding its practical administration into the wound [15]. Hence, in rabies-endemic countries, RIG is often in short supply [16] because of insufficient demand and supply forecasting. In some countries, reluctance to equine blood-derived RIG has also been noted. Solid scientific evidence exists to demonstrate the high safety and efficacy of RIG in general and equine blood-derived RIG in particular [17], which is considerably less expensive than human blood-derived RIG, hence often the only viable option in resource-limited contexts. If any RIG volume is left after administration to one patient, injecting it intramuscularly far from the wound provides no or little additional protection against rabies. The volume of RIG remaining in the vial should be better saved for another patient.

RIG should be administered as soon as possible. RIG administration requires well-informed healthcare providers. While RIG infiltration is not technically challenging, calculating the necessary RIG amount requires some practice. If a patient has ever received any rabies vaccination (PEP or PrEP) more than 7 days prior to a Category III exposure, RIG must not be given (exceptions exist for imunocompromised patients as illustrated in Fig. 1).

Healthcare providers need to understand the cost-, dose- and time-saving benefits of intradermal vaccination and have the chance to clarify any doubts or concerns that may prevent them from choosing intradermal vaccination whenever possible. Current vaccines (>2.5 IU/intramuscular dose), when administered intradermally, are equally or more efficient than when administered intramuscularly [18]. While the intradermal regimen is especially cost-effective in clinics that see several patients a day [19], it is still worth-using - and cost-effective - even in settings where only one or two patients are typically treated per day. Healthcare providers also need to be adequately trained in intradermal administration, as unintended intramuscular or subcutaneous administration (under the skin vs. in the skin) can lead to a lack of efficacy and vaccination failure. Multilingual demonstrational videos and supervised practice (through the routine adoption of intradermal vaccine administration) are key to improving this skill in all the healthcare providers who attend to patients with animal wounds in dedicated clinics but also in general healthcare facilities, especially in rural areas. Prior experience in performing the better-known Mantoux tuberculin skin test [12] is helpful.

For the intradermal route, one dose is 0.1 mL of cell culture and embryonated egg-based rabies vaccine (irrespective of the vaccine brand). All rabies vaccines can safely be used intradermally, even if this constitutes off-label use. Most vaccines, which were initially developed for intramuscular administration, provide a volume of 0.5 mL or 1.0 mL after reconstitution, depending on the type of vaccine. When used for intradermal administration, one vial can therefore be fractionated to provide PEP for up to 5 individuals. Intradermal regimens use at least 25% fewer vaccine vials than intramuscular ones. When more patients seek care at clinics on the same day, intradermal regimens become increasingly cost-effective, using up to 85% less vaccine [20]. If no patient shows up within the 6–8 h timeline before the disposal of the open vial, the vaccine remaining in the vial can be used for PrEP in animal and human health professionals at occupational risk, or for relatives or accompanying persons of exposed patients [21]. This minimizes the waste of opened – yet still shareable – vials.

## 4 Conclusion

To reach the global goal of zero human deaths from dog-transmitted rabies by 2030, everyone who is exposed to a suspected rabid animal needs to be treated efficiently according to the latest WHO recommendations. Following this protocol helps achieve a triple benefit.

First, no life is lost to this preventable disease.

Second, the benefits of intradermal vaccination over intramuscular vaccination are appreciated. Intradermal: (1) saves costs to the healthcare system because one vial can be used for more than one patient, (2) pleases the healthcare providers, because by saving vaccine doses, intradermal vaccination increases the likelihood of vaccines being always in-stock even in remote clinics, hence reduces chances of conflict with patients, and (3) pleases the patient, because only three visits to the healthcare facility are required, and vial sharing reduces the cost of the single vaccination (when the healthcare system is not able to offer it free of charge) (Table 3).

**Table 3:** Comparison of intradermal vs. intramuscular rabies vaccine administration in terms of cost, dose and time sparing.

WHO- recommended post-exposure prophylaxis regimen	Number of injection sites per visit (days 0, 3, 7, 14, 21–28)	Dose per injection (mL)	Total amount of vaccine needed (mL)	Total number of vials needed	Total number of visits needed	Days needed to complete PEP	Maximum saving of vaccine (%)
Intramuscular (IM)	1–1–1–1–0	The entire vial: 0.5 or 1 (depending on vial size)	2 or 4 (depending on vial size)	4	3–4	14–28	0
	or						
	2–0–1–0–1						
Intradermal (ID)	2–2–2–0–0	0.1	0.6	∼1	3	7	60–80

Third, doctor-patient communication and community engagement are strengthened, to increase the compliance of the patient in question as well as the likelihood that other members of their community will seek PEP in case of future exposure.

## Ethical approval statement

This paper required no ethical approval.

## Funding source

This paper received no specific grant from any funding agency.

## Declaration of Competing Interest

The authors declare that they have no known competing financial interests or personal relationships that could have appeared to influence the work reported in this paper.
