# Supplementary material for: Rabies post-exposure prophylaxis delivery to ensure treatment efficacy and increase compliance
Source: IJID One Health. 2023 Dec;1:100006. doi: 10.1016/j.ijidoh.2023.100006 (PMC10752235; doi:10.1016/j.ijidoh.2023.100006)
Supplement: Supplementary file 1 — Supplementary material [file mmc1.pdf]

PROTOCOL FOR A WELL-PERFORMED RABIES POST-EXPOSURE PROPHYLAXIS DELIVERY

1. Wound treatment

Thorough wound washing is a life-saving measure to mechanically reduce the viral inoculum at the wound site and is crucial in all exposures but especially when the patient's trip to the healthcare facility is long or no rabies biologicals are readily available.

1.1. Wash and flush the wound (or all wounds, if more than one) with copious amounts of water (preferably running water, if available) and soap or detergent for 15 min.

1.1.1. ***If*** eyes or mucosa were exposed, thoroughly rinse them with water.

1.2. Apply an antiseptic (e.g., povidone-iodine) thoroughly on the wound.

1.3. Since the wound is likely contaminated with dirt or soil, administer tetanus vaccination to the patient, either as a primary series or a booster if the last dose was received more than 10 years before (or as per the national vaccination guidelines). Consider broad-spectrum antibiotics to prevent bacterial infection especially in deep wounds, and follow the dosage recommended by the manufacturer.

1.4. Recommend the patient against applying home-based remedies to the wound.

1.5. Do not close or tightly cover the wound with dressings or bandages and recommend the patient against doing this.

1.6. Avoid suturing. If not possible (e.g., continued bleeding, risk of visible scar), try to delay it at least for some hours after the administration of RIG/RmAbs (if this rabies biological is necessary) to allow its infiltration through the tissues. If suturing cannot be delayed, sutures should be loose and minimal.

2. Risk evaluation and assessment of the need for rabies biologicals

2.1. Check the severity of the wound.

2.1.1. Assess the number of wounds and their depth, and determine the WHO wound category:

- ***Category I:*** The skin is still intact (test: the patient feels no burning sensation when surgical spirit is applied on intact skin): animal licks on intact skin, touching or feeding animals. This is not an exposure.
- ***Category II:*** The skin is broken but there is no bleeding (test: the patient feels a burning sensation when surgical spirit is applied on broken skin): minor scratches or abrasions, nibbling of uncovered skin. This is a mild exposure.
- ***Category III:*** The skin is broken and the wound is bleeding, mucous membrane or broken skin has been contaminated with saliva or direct contact with wild animals (including bats) has occurred. This is a severe exposure.

2.2. Consider the anatomical position of the wound.

2.2.1. Assess if it is a wound to a body part that is close to the brain or highly innervated, hence making it easier and faster for the virus to reach the brain. Such high-risk areas are the head, neck, face, genitals, or hands.

2.3. Assess if the patient is immunodeficient.

2.3.1. Ask the patient whether they suffer from any immune system disorder, or are HIV positive and on any anti-retroviral treatment, or are on long-term steroids or anti-cancer drugs.

2.4. Assess the vaccination history of the patient.

2.4.1. Ask the patient whether they have ever received any rabies vaccination (preferably with proof of vaccination, e.g., vaccination card), either before any exposure (as pre-exposure prophylaxis, PrEP) or after any exposure (as PEP).

2.4.2. ***If*** the patient has ever received any rabies vaccination before, ask the patient how many doses they have received. At least two doses of a cell culture vaccine received on an appropriate schedule before discontinuation count as PrEP. If the patient cannot remember the number of doses received or vaccination dates, or cannot show proof of prior vaccination, consider them unvaccinated patients.

2.4.3. ***If*** the patient has ever received any rabies vaccination before, ask the patient when they have received these doses. If complete PEP has been received in the last 3 months, immediate vaccination is not recommended. Since the patient might not know for sure whether they received the rabies vaccine, or another vaccine, proof of prior vaccination is recommended.

2.4.4. ***If*** the patient has ever received any rabies vaccination before, ask the patient whether they had any adverse events after vaccination. If the patient had a mild local reaction (e.g., pain, redness or swelling), they can continue PEP using the same brand of rabies vaccine. If they had a severe local or systemic reaction, another type of rabies vaccine should be used, if available (e.g., purified chick embryo cell vaccine, purified vero cell rabies vaccine).

2.5. Gather information from the patient on the animal involved in the exposure.

2.5.1. Ask the patient what animal they were exposed to and determine whether it's a rabies-susceptible animal. Only mammals are susceptible to rabies, although to different extents (e.g., rabies is rare in rodents, and no rodent bite is known to have caused a human rabies death).

2.5.2. Ask the patient to give you any further information about the animal and the exposure to it (**Table 1**).

2.6. Use all the information obtained to decide whether rabies vaccines and RIG/RmAbs are needed right away, can be delayed or are not applicable. For guidance on wound categorization and assessing the animal status, decision trees can be consulted (**Figure 1 – Wound risk assessment** and **Figure 2 – PEP risk assessment**). For example, if the patient has ever received any rabies vaccination (PEP or PrEP) more than 7 days prior to the new Category III exposure, RIG must not be given.

2.7. Fully and clearly explain to the patient the provided treatment (including any injection other than rabies biologicals, to avoid confusion in the patient) and the proposed rabies vaccination schedule, specifying how many visits and injections will be necessary (**Figure 3**). Whenever possible, use the local language and choose words that are understandable to illiterate patients.

2.8. Obtain consent to proceed as per local law and regulations.

Table 1: Questions for patients about the animal involved and the exposure to it

- When did exposure occur?
- Is the animal still alive?
  - If no, when did it die?
- Was the animal tested for rabies?
  - If yes, what is the result of the test?
  - If no, is the animal available for testing or quarantine?
- Do you know for sure whether the animal was vaccinated against rabies?
- How did the bite/scratch/exposure happen? What was the animal doing and what were you doing?
- Did the animal show any of the following symptoms like:
  - Hypersalivation?
  - Paralysis?
  - Lethargy?
  - Abnormal aggression (e.g., biting two or more people or animals and/or inanimate objects)?
  - Strange vocalization?
  - Diurnal activity (in case of nocturnal species)?
- Has the animal scratched/bitten other people/animals?
  If yes, inform them about the need for PEP, through local human and animal healthcare providers.

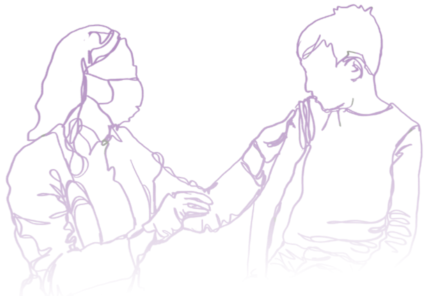

Wound risk assessment

Animal: mammal?
- N → Species not susceptible for rabies: no PEP indicated, wash exposed areas with soap and water
- Y → Animal: rodent?
  - Y → Rodents rarely transmit rabies: no PEP indicated, wash exposed areas with soap and water
  - N → Mucous membrane: contaminated with saliva?
    - Y → Category III
    - N → Skin: broken?
      - N → Category I
      - Y → Animal: bat?
        - Y → Category III
        - N → Wound: bleeding?
          - Y → Category III
          - N → Category II

ARV = Anti Rabies Vaccine
PEP = Post-Exposure Prophylaxis
RIG = Rabies Immunoglobulin
RmAbs = Rabies Monoclonal Antibodies
Y = Yes, N = No

Category I

WHO wound category I

Animal licks on intact skin, touching or feeding the animal

No PEP indicated, wash exposed areas with soap and water

Category II

WHO wound category II

Minor scratches or abrasions, nibbling of uncovered skin

Patient: immunocompromised?
- Y → Wound washing[1], RIG/RmAbs and ARV immediately[2]
- N → Patient: ever received any rabies vaccination?[3]
  - N → Wound washing[1] and ARV → Proceed with PEP Risk Assessment[6]
  - Y → Patient: completed PEP less than 3 months ago?
    - Y → No PEP indicated, wash exposed areas with soap and water
    - N → Wound washing[1] and ARV[4] → Proceed with PEP Risk Assessment[6]

Category III

WHO wound category III

Transdermal bites or scratches, licks on broken skin, contamination of mucous membrane with saliva, exposure to a bat

Patient: immunocompromised?
- Y → Wound washing[1], RIG/RmAbs and ARV immediately[2]
- N → Patient: ever received any rabies vaccination?[3]
  - Y → Patient: completed PEP less than 3 months ago?
    - Y → No PEP indicated, wash exposed areas with soap and water
    - N → Wound washing[1] and ARV[4]
  - N → RIG/RmABs availability: limited?
    - N → Wound washing[1], RIG/RmAbs and ARV → Proceed with PEP Risk Assessment[6]
    - Y → Patient: high-risk?[5]
      - Y → Wound washing[1], RIG/RmAbs and ARV → Proceed with PEP Risk Assessment[6]
      - N → Wound washing[1] and ARV → Proceed with PEP Risk Assessment[6]

1 Wash the wound thoroughly with copious amounts of water and soap for 15 min and apply an antiseptic. This is a life-saving practice especially for immunocompromised patients.
2 When feasible, the RABV neutralizing antibody response should be determined 2–4 weeks after vaccination to assess whether an additional dose of vaccine is required.
3 This includes Pre-Exposure Prophylaxis (PrEP), previous PEP, or patients beyond the 7th day of PEP.
4 If PrEP or previous PEP was received: shorten the current vaccination schedule accordingly.
5 Patient with multiple bites, deep wounds, bites to highly innervated parts of the body (such as head, neck, face, genitals and hands), severe immunodeficiency, bites from an animal with probable (clinically) or confirmed (laboratory) rabies, exposure to a bat (bite, scratch or exposure of mucous membrane).
6 If your program does not have adequate surveillance to assess the biting animal (as recommended in the PEP Risk Assessment Decision Tree), PEP should be initiated immediately.

PEP risk assessment

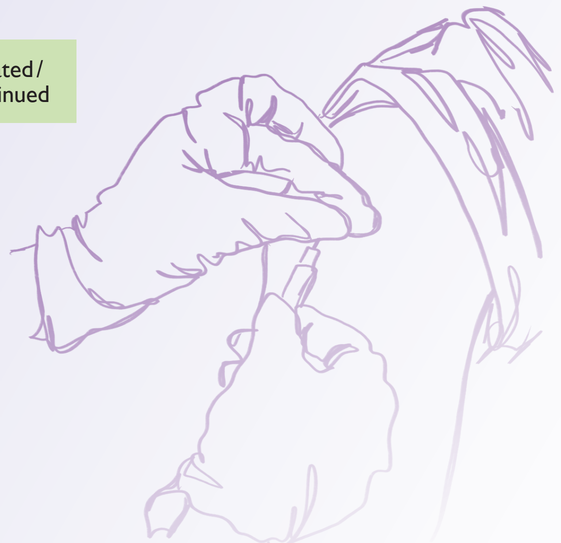

Did the exposure occur >10 days ago?
- Y → Is the animal still alive?[1]
  - Y → PEP not indicated / can be discontinued
  - N/U → Is the victim <14 years? …
- N → Is the victim <14 years?

Is the victim <14 years?
OR
Does the bite victim have multiple deep wounds?
OR
Does the bite victim have bites to highly innervated areas?[2]
- Y → Start PEP immediately
- N → Has the animal been tested for rabies?

Has the animal been tested for rabies?
- Y →
  - Test positive → Start PEP immediately
  - Test pending / inconclusive → Start PEP immediately
  - Test negative → PEP not indicated / can be discontinued[4]
- N/U → Did the animal show any signs of disease consistent with rabies? …

Did the animal show any signs of disease consistent with rabies?[3]
OR
Did the animal die within 10 days of the exposure?[1]
OR
Did the animal bite 2 or more people or animals?
- Y/U → Is the animal still alive?
  - N/U → Start PEP immediately
  - Y → Is the animal up-to-date on vaccination?
    - N/U → Start PEP immediately
    - Y → Was the animal exposed to rabies risk in the last 12 months?
      - Y/U → Start PEP immediately
      - N → Is the animal available for assessment and quarantine?
        - N/U → Start PEP immediately
        - Y → Delay PEP pending assessment and quarantine results[1]
- N → Is the animal available for assessment and quarantine?

Is the animal available for assessment and quarantine?
- N/U → Start PEP immediately
- Y → Is the animal up-to-date on vaccination?

Is the animal up-to-date on vaccination?
- N/U → Was the animal provoked to bite /scratch?
  - Y → PEP not indicated / can be discontinued[4]
  - N/U → Start PEP immediately
- Y → Was the animal provoked to bite/scratch?

Was the animal provoked to bite/scratch?
- N/U → Delay PEP pending assessment and quarantine results[1]
- Y → PEP not indicated / can be discontinued[4]

PEP = Post-Exposure Prophylaxis
Y = Yes, N = No, U = Unknown

1 Dogs, cats and domestic ferrets being healthy 10 days post-exposure present no rabies risk and PEP is not indicated. The infectious periods of other animals are not well characterized, and a more conservative 14-day clinical investigation is recommended.

2 Such as bites to the head, neck, face, genitals and hands.

3 Clinical signs of rabies in an animal include hypersalivation, paralysis, lethargy, unprovoked abnormal aggression (e.g. biting two or more people or animals and/or inanimate objects), abnormal vocalization, diurnal activity of nocturnal species. Hydrophobia is not a sign of rabies in dogs.

4 This risk assessment is made at one point in time with the available information. If new information is provided or the status of the animal changes, PEP might be indicated.

3. Administration of RIG/RmAbs

The timely administration of RIG, or the most recent RmAbs, provides passive immunization by neutralizing the rabies virus at the wound site before the immune system responds to the vaccine. Therefore, the products need to be administered into and around all wounds. RIG is derived from human blood (hRIG) or equine blood (eRIG). Strong evidence shows that both RIG versions have similar efficacy. In clinical trials, several RmAb products against rabies have proved to be safe and effective in neutralizing a broad panel of globally prevalent rabies virus isolates. Advantages of RmAb products include large-scale production with standardized quality, high effectiveness and reduced risk of adverse events. RIG and RmAb products that are licensed for use in humans should be prioritized. WHO has recommended use of mAb "cocktails" containing at least two antibodies against the rabies virus.

3.1. Take the RIG/RmAb box out of a properly functioning refrigerator.

3.2. Calculate the maximum amount of RIG/RmAbs that the patient could receive: 20 IU (international unit)/kg of body weight for hRIG, 40 IU/kg of body weight for eRIG, 3.33 IU/kg of body weight for single RmAb and 40 IU/kg body weight for cocktail RmAbs.

3.2.1. ***If*** the wound is small, estimate the maximal quantity that is anatomically feasible to infiltrate and ensure not to exceed it.

3.3. Draw the needed amount of RIG/RmAbs into a new syringe.

3.4. Fractionate the rest into smaller, individual syringes to be used for other patients.

3.5. Dilute RIG/RmAbs for large and multiple wounds with sterile normal saline, if needed.

3.6. ***For RIG:*** infiltrate the entire necessary amount or as much as possible carefully deep into or as close as possible to all wound(s) or exposure sites, avoiding any compartment syndrome. Injecting the remaining RIG volume intramuscularly at a distance from the wound provides no additional protection against rabies (see exception below). ***For RmAbs:*** follow the manufacturer's instructions.

3.6.1. ***If*** there is a high likelihood that there are additional small wounds (e.g., if a child does not report all wounds), exposure was to bats, or exposure was other than through a scratch or bite, inject the remaining RIG volume intramuscularly <u>as close as possible</u> to the presumed exposure site, to the degree that is anatomically feasible.

3.6.2. ***If*** exposure was in the mucosa, rinse the exposed part with RIG.

3.6.3. ***If*** exposure was via aerosols (e.g., in a laboratory), inject RIG intramuscularly.

3.7. Observe the patient for 15–20 min after the administration for potential adverse events, while you continue with the next steps. ERIG should be administered under conditions that allow the management of an anaphylactic reaction (i.e., a severe, potentially life-threatening allergic reaction to a substance such as a vaccine component). Nevertheless, the risk for an anaphylactic reaction is low (1/150,000), and the reaction is generally treatable.

3.8. Store open vials and draw up doses (using new needles) if other patients come in on the same day.

3.9. Discard unused vials and doses at the end of the day.

4. Administration of rabies vaccine

Since 1984, WHO has strongly suggested the discontinuation of old-fashioned nerve tissue vaccines and their replacement with modern, concentrated, purified cell culture and embryonated egg-based rabies vaccines (CCEEVs). Modern rabies CCEEVs are safe and well tolerated and should comply with the recommended potency of ≥ 2.5 IU per vial. All vaccines can be used both for intramuscular administration (i.e., injection in the muscle) and intradermal administration (i.e., injection in the upper layer of the skin). For dose-, cost- and time-saving reasons, WHO recommends intradermal administration in a shortened, 1-week vaccination schedule (**Figure 3**). Rabies vaccines are safe to be used intradermally even when they are only labelled for intramuscular use (off-label use).

4.1. ***If*** available, use WHO-prequalified cell culture vaccines (an updated list is available on the WHO website). If WHO-prequalified cell culture vaccines are not available, use the cell culture vaccines recommended in national guidelines.

4.2. Take the rabies vaccine box out of a properly functioning refrigerator. Rabies vaccines must be refrigerated at 2–8 °C, kept away from sunlight, and not stored in the refrigerator door (because the temperature may fluctuate when opening and closing it). The temperature of the refrigerator needs to be monitored and adjusted as necessary, especially in case of power cuts.

4.2.1. ***If*** an unopened vial is used, reconstitute the rabies vaccine according to the manufacturer's instructions. All rabies vaccines are lyophilized and need reconstitution before use with the accompanying sterile diluent. Shake the vial 2–3 times before withdrawing the vaccine into the syringe.

4.2.2. ***If*** an already opened vial is used, check if it was stored hygienically and opened less than 6–8 h before. No need for reconstitution is there. Rabies vaccines must be used immediately after dilution, or within 6–8 h only if stored at 2–8 °C and protected from sunlight. Shake the vial 2–3 times before withdrawing the vaccine into the syringe.

4.3. Draw up 0.2 mL of rabies vaccine with an insulin syringe.

4.4. Inject 0.1 mL of vaccine intradermally in the deltoid area for adults and the anterolateral area of the thighs for young children aged < 2 years. Rabies vaccines should never be administered in the gluteal area because this results in lower neutralizing antibody titres and should never be administered in the same anatomical site as RIG/RmAbs.

4.4.1. Insert the needle into the upper layer of the skin, with the bevel facing upwards, at a 45-degree angle and approximately 2 mm into the skin (similar to the Mantoux tuberculin skin test).

4.4.2. Start injecting and notice whether you feel any resistance. If not, the needle may wrongly be in the subcutaneous tissue. In this case, withdraw the needle and repeat the injection in a new site.

4.4.3. Inject 0.1 mL until you see a small (i.e., 6-8 mm in diameter) bleb with an "orange peel" appearance (**Figure 4**).

4.4.4. Do not rub the injection site.

4.5. Repeat the same on the other arm.

4.5.1. ***If*** the wound(s) is on an arm, the vaccine should be administered intradermally in the anterolateral area of the thighs or the suprascapular areas.

4.6. Safely dispose of the used syringe and needle.

4.7. Observe the patient for 15–20 min after the administration for potential adverse events.

4.8. Store the opened vial hygienically.

4.9. Hand out a rabies-specific vaccination card and remind the patient about the follow-up visits to complete PEP.

4.10. If the patient knows of any other person who was exposed to the same animal, ask the patient to promptly inform them about where to go to receive PEP.

4.11. Register the information about exposure and treatment for national surveillance. The number of reported animal exposure cases in humans and the number of people receiving PEP (disaggregated by sex, age, species of exposed animal, and WHO exposure category) are key rabies indicators that need to be part of any national rabies surveillance systems.

4.12. After 6–8 h from opening the vial, dispose of it.

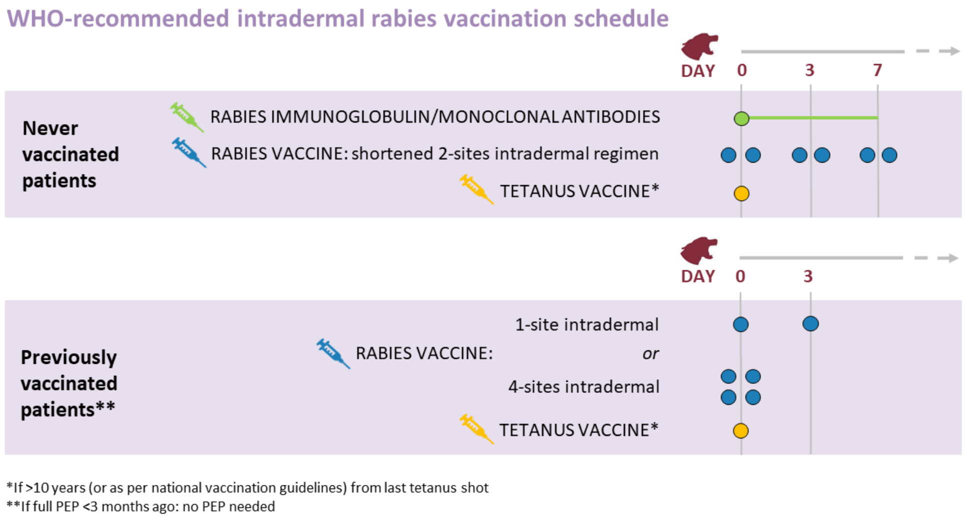

Figure 3: WHO-recommended 1-week intradermal post-exposure prophylaxis vaccination schedule. For exposed individuals who have not been previously immunized, WHO recommends a 1-week vaccination schedule on days 0, 3 and 7, with a 2-site intradermal injection on each day.

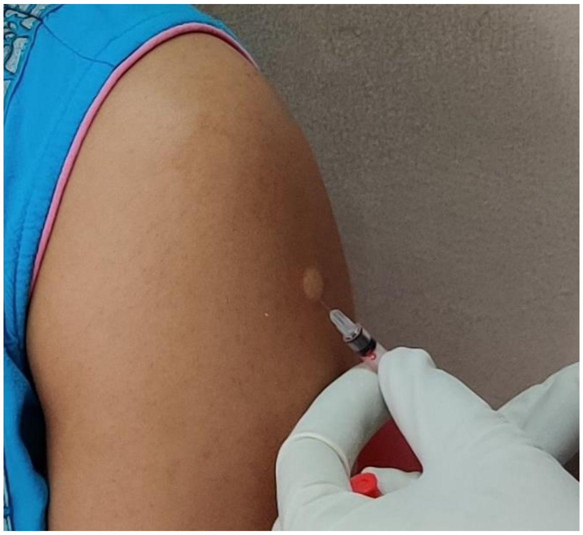

Figure 4: Confirmation of correct intradermal rabies vaccine administration. When the rabies vaccine is correctly injected into the upper skin area (over the deltoid muscle in adults or the lateral thigh area of infants), a distinctive bleb with an 'orange peel' appearance is visible.

5. Patient counselling

5.1. Get ready to answer the questions that your patients may have (**Table 2A**).

5.2. Get ready to answer the questions that the less experienced colleagues may have (**Table 2B**).

Table 2A: Questions frequently asked by patients

I was scratched/bitten a long time ago. Do I still need rabies vaccination?	Yes, because the rabies incubation period can be very long.
If I start rabies vaccination, will I need to change my diet?	No, you can eat whatever you want.
Will you give me many injections in the stomach?	No, that was done with the outdated nerve tissue vaccine (NTV). Modern vaccines only require a few doses and a normal injection in the arm, as with any other injectable vaccine.
Will there be adverse effects?	As with any vaccination, there may be adverse effects. But they are likely to be minor (such as redness, pain or swelling at the site of injection) or, even unlikely, mild (such as some fever, headache, dizziness or gastrointestinal symptoms). Serious adverse effects like allergic reactions are rare.
What happens if I forget to come for the next vaccination?	Make sure you don't forget by having your relatives/friends remind you about it. But if you forget, come as soon as possible and we will continue the vaccination, not restart it.
I had milk from a rabid animal. Do I need rabies vaccination?	No, you don't need any PEP, but avoid it next time and, anyway, milk should best be boiled before consumption.
I had meat from a rabid animal. Do I need rabies vaccination?	No, you don't need any PEP, but avoid it next time and, anyway, meat should best be cooked before consumption.
I processed the meat of a rabid animal. Do I need rabies vaccination?	Probably yes. Tell me more.
I was bitten by a rat. Do I need rabies vaccination?	No, there is no risk of rabies.
I am pregnant. Is rabies vaccination safe for me and my baby?	Yes.
I am breastfeeding. Is rabies vaccination safe for my baby?	Yes.

Table 2B: Questions frequently asked by healthcare providers

Does the dose of rabies vaccine depend on age or weight?	No. Age only determines the site of rabies vaccination: the deltoid area for adults and the anterolateral area of the thighs for children <2 years. Weight only determines the maximum amount of RIG/RmAbs to use: 20 IU/kg of body weight for hRIG, 40 IU/kg of body weight for eRIG, 3.33 IU/kg body weight for single mAb and 40 IU/kg body weight for cocktail mAb.
Can I change the administration route or vaccine product during the vaccination schedule?	Yes, if unavoidable, you can do it. Don't restart vaccination, just continue it.
How does intradermal vaccination work when the dose is so small?	The antigen-presenting cells in the dermis are more effective in presenting the vaccine/antigen to the immune system than the ones in the muscles, so they can stimulate a very strong and prompt immunologic/antibody response.
The rabies vaccine box only mentions intramuscular vaccination. Can I administer the vaccine intradermally?	Yes, all vaccines can be given both intramuscularly and intradermally, but choose the intradermal route whenever possible because it's cheaper for the healthcare system and the patient, requires fewer visits, and one vaccine vial can be shared across many patients. However, check the rabies vaccines that your national drug regulatory authority approves for intradermal use.
Can I inject the rabies vaccine in the gluteal area?	No, the vaccine would not be fully absorbed and effective because of the fat present in that body part.
If the wound(s) is on an arm, where should I inject the rabies vaccine?	You should inject the vaccine intradermally in the anterolateral area of the thighs or the suprascapular areas. RIG must be injected in the wound(s).
If RIG/RmAbs is not available on day 0, should I delay rabies vaccination?	No, never. But refer the patient to a healthcare facility where RIG/RmAbs is available, after administering the first dose of rabies vaccine.
Can I administer RIG/RmAbs later on in the vaccination schedule?	Yes, if RIG/RmAbs is not available on day 0, but never after day 7. Anyway, RIG/RmAbs should be given as soon as possible after exposure.
Can I give RIG/RmAbs to a patient who has already received any rabies vaccination in their lifetime?	No, thanks to the previous vaccination, there are already demonstrable antibody titres or immune memory cells. In case of re-exposure, 1-site intradermal rabies vaccine administration on days 0 and 3 *or* 4-sites intradermal rabies vaccine administration on day 0 will produce good antibodies due to anamnestic response.
Should I perform a skin test before administering eRIG?	No, because they poorly predict severe adverse events and their results must anyway not be the reason for not giving eRIG if it is needed. However, all RIG should be administered under conditions that would allow management of an anaphylactic reaction.
Can I give rabies biologicals to a patient who is receiving treatment with chloroquine or hydroxychloroquine?	Yes, given the fatal outcome of rabies, there is no contraindication to the concomitant use of any medication.
Can I give rabies biologicals to a patient who is receiving other vaccines in this period?	Yes, given the fatal outcome of rabies, priority is given to rabies biologicals (rabies vaccine and RIG/RmAbs). If the patient receives RIG, live vaccines should be postponed for 3-4 months, if possible.
Should I perform an antibody test on the patient following rabies vaccination?	No, unless the patient is immunocompromised.

Are there special recommendations for patients undergoing chemotherapy?	Yes, they are to be treated as immunocompromised patients. So: emphasis on proper wound washing; immediate RIG/RmAbs and rabies vaccine, even if previously immunized, for category-II and -III exposure; complete rabies vaccination course; Rapid Fluorescent Foci Inhibition Test 2–4 weeks after vaccination.
Are all HIV-infected individuals considered immunocompromised?	No. HIV-infected individuals who receive antiretroviral therapy and are clinically well and immunologically stable (i.e., normal CD4% > 25% for children aged < 5 years or CD4 cell-count ≥ 200 cells/mm³ if aged ≥5 years) are not considered immunocompromised.
Can intradermal administration be used for immunocompromised individuals or individuals receiving chloroquine, hydroxychloroquine drugs or long-term corticosteroid or other immunosuppressive therapy?	Yes.
